# Supplementary material for: Rap1 Is Involved in Angiopoietin-1-Induced Cell-Cell Junction Stabilization and Endothelial Cell Sprouting
Source: Cells. 2020 Jan 8;9(1):155. doi: 10.3390/cells9010155 (PMC7016689; doi:10.3390/cells9010155)
Supplement: Supplementary file 1 [file cells-09-00155-s001.pdf]

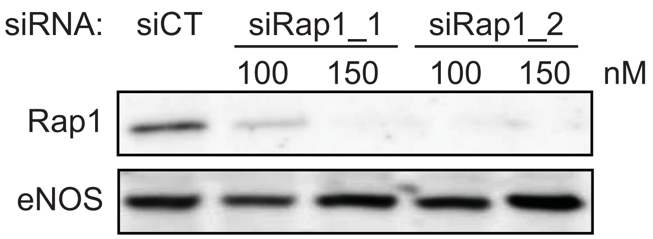

Figure S1. Validation of Rap1 siRNAs. BAECs were transfected with control siRNA (siCT) or with two distinct siRNAs targeting Rap1 (siRap1_1 and siRap1_2) and downregulation of Rap1 was confirmed by immunoblotting against Rap1. Western blot against eNOS was used as a loading control.
